# Identification and biochemical characterisation of *Acanthamoeba castellanii* cysteine protease 3

**DOI:** 10.1186/s13071-020-04474-8

**Published:** 2020-11-23

**Authors:** Zhixin Wang, Duo Wu, Hiroshi Tachibana, Meng Feng, Xun-jia Cheng

**Affiliations:** 1 Department of Medical Microbiology and Parasitology, School of Basic Medical Sciences, Fudan University, Shanghai, 200032 China; 2 Department of Infectious Diseases, Tokai University School of Medicine, Isehara, Kanagawa 259-1193 Japan

**Keywords:** *Acanthamoeba castellanii*, Cysteine protease, Virulence factor, Encystment, p53 pathway

## Abstract

**Background:**

*Acanthamoeba* spp. are free-living amoeba that are ubiquitously distributed in the environment. This study examines pathogenic *Acanthamoeba* cysteine proteases (*Ac*CPs) belonging to the cathepsin L-family and explores the mechanism of *Ac*CP3 interaction with host cells.

**Methods:**

Six *Ac*CP genes were amplified by polymerase chain reaction (PCR). Quantitative real-time PCR was used to analyse the relative mRNA expression of *Ac*CPs during the encystation process and between pre- and post-reactivated trophozoites. To further verify the role of *Ac*CP3 in these processes, *Ac*CP*3* recombinant proteins were expressed in *Escherichia coli*, and the hydrolytic activity of *Ac*CP*3* was determined. The influence of the *Ac*CP3 on the hydrolytic activity of trophozoites and the toxicity of trophozoites to human corneal epithelial cells (HCECs) was examined by inhibiting *Ac*CP3 expression using siRNA. Furthermore, the levels of p-Raf and p-Erk were examined in HCECs following coculture with *Ac*CP3 gene knockdown trophozoites by Western blotting.

**Results:**

During encystation, five out of six *Ac*CPs exhibited decreased expression, and only *Ac*CP*6* was substantially up-regulated at the mRNA level, indicating that most *Ac*CPs were not directly correlated to encystation. Furthermore, six *Ac*CPs exhibited increased expression level following trophozoite reactivation with HEp-2 cells, particularly *Ac*CP3, indicating that these *Ac*CPs might be virulent factors. After refolding of recombinant *Ac*CP3 protein, the 27 kDa mature protein from the 34 kDa pro-protein hydrolysed host haemoglobin, collagen and albumin and showed high activity in an acidic environment. After *Ac*CP3 knockdown, the hydrolytic activity of trophozoite crude protein against gelatin was decreased, suggesting that these trophozoites had decreased toxicity. Compared with untreated trophozoites or negative control siRNA-treated trophozoites, *Ac*CP3-knockdown trophozoites were less able to penetrate and damage monolayers of HCECs. Western blot analysis showed that the activation levels of the Ras/Raf/Erk/p53 signalling pathways in HCECs decreased after inhibiting the expression of trophozoite *Ac*CP3.

**Conclusions:**

*Ac*CP6 was correlated to encystation. Furthermore, *Ac*CP3 was a virulent factor in trophozoites and participated in the activation of the Ras/Raf/Erk/p53 signalling pathways of host cells. 
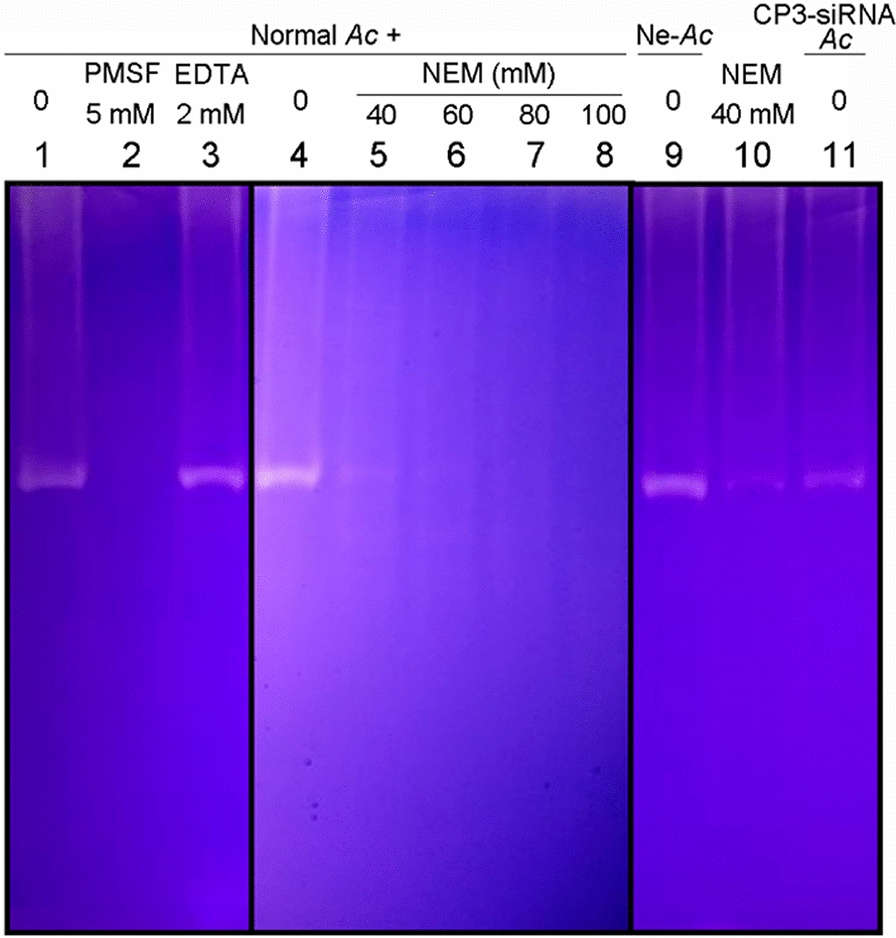

## Background

*Acanthamoeba castellanii* is a free-living amoeba that can cause Acanthamoeba keratitis in humans after infection as well as granulomatous amoebic encephalitis and cutaneous acanthamoebiasis in immunocompromised individuals [1, 2]. In recent years, serious *Acanthamoeba* infections have been associated with an increasing number of contact lens wearers and immunocompromised patients. This parasite has a life cycle with two phases, the trophozoite and the cyst, which differ morphologically. When exposed to starvation conditions or a change in osmotic pressure, the trophozoites transform into cysts to better withstand harsh environmental conditions. This parasite readily encysts in response to nutrition source deprivation, osmotic shock or a combination of both [3]. Thus, the growth and encystation of *Acanthamoeba* trophozoites, which are regulated by a number of biological factors, can lead to persistent infections and influence the pathogenicity of this parasite.

The first stage of *Acanthamoeba* infection is the interaction between mannose binding protein (MBP) on the surface of the parasite and the mannosylated proteins on the surface of host cells [4]. Next, a variety of proteolytic enzymes are secreted into the host, hydrolysing the host’s tissue and resulting in tissue oedema, inflammation and necrosis [5]. Among these enzymes, serine proteases [6] and cysteine proteases (CPs) [7] are the main proteolytic proteins. Studies have shown that many serine proteases with different molecular weights are involved in the degradation of host cells and extracellular matrix [8] during pathogenic *Acanthamoeba* infection and are inhibited by proteasome inhibitors in a concentration-dependent manner [6].

Many protozoa utilise CPs for nutrient uptake, host infection, tissue invasion and environmental adaptation (encystation). It was well established that pathogenicity-related CPs, including cysteine protease 1 (CP1), CP2 and CP5, are expressed during the pathogenic process of *Entamoeba histolytica* [9]. In *Giardia lamblia*, CPs and UDP-N-acetylglucosamine pyrophosphorylase are required during encystation. *Acanthamoeba* expresses many CPs with different molecular weights, including 38.5, 43, 50, 59, 70, 100 and 130 kDa proteases [10–13]. After sequencing and analysis, it was found that both the 990 bp *A. castellanii* CP gene (*Ac*CP) [11] and the 1359 bp *A. culbertsoni* CP gene (*Ac*CP2) [14] belong to the L cathepsin CP family. *Ac*CP2 contains an Ex3Rx3Wx2N motif in the proregion and a proline/threonine-rich C-terminus. The amino acid sequence of *Ac*CP contains a catalytic site with five residues as well as ERFNIN and GNFD motifs. Moreover, recombinant *Ac*CP protein was capable of hydrolysing host proteins, including haemoglobin, albumin, IgG, IgA and adhesion proteins, suggesting that *Ac*CP may be an important pathogenic protease in *Acanthamoeba* [11].

The pathogenic process of *Acanthamoeba* mainly depends on the hydrolytic activity of the pathogenic proteases secreted by trophozoites, which activate a series of cell signalling pathways in host cells. Several studies have revealed that various downstream molecules are involved, including G-protein-coupled receptors, beta adrenalin receptors [15], Toll-like receptor-4 (TLR4), TLR4-myeloid differentiation primary response gene 88 (MyD88), nuclear factor-κB (NF-κB), extracellular signal-regulated kinase (Erk) [16], phosphatidylinositol-3-kinase (PI3K) [17] and cytosolic phospholipase (A2αcPLA2α) [18]. Due to the complex pathogenic mechanisms of *Acanthamoeba* infection, cytopathogenic effects in host cells are not solely mediated by these signalling molecules. Instead, changes in the expression levels of other signalling molecules in the host may be involved. The Ras/Raf/Erk signalling pathway is well known as a key intracellular signal transduction pathway regulating cell differentiation, proliferation and apoptosis and can be regulated by various growth factors. When cells are stimulated, Ras protein kinase expression is up-regulated, thereby activating downstream Erk1/2 protein. Furthermore, the activation of Ras/Raf/ERK signalling can lead to the activation of the tumour suppressor p53. Increased phosphorylation of p53 can up-regulate apoptotic proteins and inhibit bcl-2 proteins, eventually inducing apoptosis [19]. However, whether the Ras/Raf/Erk/p53 signalling pathways of host cells are involved in the pathogenesis of *Acanthamoeba* has not yet been verified. In addition, it has not been reported whether the CPs secreted by trophozoites are involved in the activation of the Ras/Raf/Erk/p53 signalling pathways of host cells.

Here, we amplified the CP genes *of A. castellanii* and determined their roles in the pathogenesis and encystation of trophozoites. We confirmed that *Ac*CP3 is specifically required for the pathogenicity and virulence of trophozoites. By potentially activating the Ras/Raf/Erk/p53 signalling pathway of host cells, *Ac*CP*3* may play an important role in the pathogenesis of *Acanthamoeba* and may be involved in the pathophysiology of *Acanthamoeba* infection.

## Methods

### Amoeba and cell culture

*A. castellanii* (American Type Culture Collection, ATCC 30011), which was originally isolated from a keratitis patient, was cultured axenically in peptone-yeast-glucose (PYG) medium at 26 °C, and trophozoites were harvested in the logarithmic growth phase. Human larynx epidermoid carcinoma cells (HEp-2) purchased from ATCC were cultured in EMEM (Gibco, USA) supplemented with 10% fetal bovine serum (FBS) (Thermo Fisher Scientific, USA), 100 U/mL penicillin and 100 μg/mL streptomycin. Human corneal epithelial cells (HCECs) purchased from Bioleaf (Shanghai, China) were grown in DMEM (Gibco, USA) with 10% FBS, 100 U/mL penicillin and 100 μg/mL streptomycin. Both cells were cultured in a 37 °C incubator with 5% CO_2_.

### Encystation assays

Encystation assays were performed as described previously [20] with slight modifications. Briefly, trophozoites from post-logarithmic growth phase cultures were treated with phosphate-buffered saline (PBS; containing 137 mM NaCl, 2.7 mM KCl, 10 mM Na_2_HPO_4_, 1.8 mM KH_2_PO_4_) with 50 mM MgCl_2_ and 10% glucose in culture plates at 30 °C (5 × 10^5^ trophozoites per mL of medium) for 72 h. The treated materials were collected at 24 h, 48 h and 72 h for RNA extraction, respectively.

### Transmission electron microscopy

Transmission electron microscopy was performed to observe the morphology of cysts. The cyst pellets were fixed in 3% glutaraldehyde and 4% formaldehyde in phosphate-buffered saline (0.1 M, pH 7.2) at 4°C for 72 h, washed three times in 0.1 M PBS (pH 7.2), postfixed with 1% osmium tetroxide in PBS at 4 °C for 2 h, washed three times in 0.1 M PBS (pH 7.2), dehydrated in an ascending ethanol series and embedded with epoxy resin 618. Ultrathin sections were cut on an LKB Ultratome control unit, stained with 3% uranyl acetate and lead citrate, and observed under a Tecnai G2 Spirit TWIN transmission electron microscope (FEI, Czech Republic).

### Cloning of *Ac*CP genes and expression level analysis

The CP genes of *Acanthamoeba* were cloned from the cDNA of trophozoites using the primers listed in the Additional file 1: Table S1 by polymerase chain reaction (PCR). PCR was performed in a 9902 Veriti 96-well Thermal Cycler (Applied Biosystems, USA) (94 °C for 3 min; 35 cycles of 94 °C for 15 s, 55 °C for 30 s and 72 °C for 1 min; followed by 72 °C for 7 min). The amplified PCR products were purified and ligated into a pMD19-T vector (Takara, Japan), and the nucleotide sequences were obtained by automated sequencing.

*A.castellanii* mRNA was extracted from trophozoites and cysts using RNeasy^®^ Plus Mini Kit (Qiagen, Hilden, Germany), and cDNA was synthesised using a PrimeScript^®^ 1st strand cDNA synthesis kit (Takara, Japan). Quantitative real-time PCR (qRT-PCR) was carried out in a final reaction volume of 20 μL according to the manufacturer’s recommendations on an ABI 7500 Real-time PCR system (Applied Biosystems, USA). Reactions were performed in a 96-well plate with TB Green Premix Ex Taq II (Takara, Japan) to analyse the expression level of *Ac*CPs. The primers for *Ac*CPs genes and the GAPDH internal reference are listed in Additional file 1: Table S2. The amplification cycling conditions were as follows: 30 s at 95 °C and 40 cycles of 5 s at 95 °C and 35 s at 60 °C. Each experiment was performed at least three times.

### Expression, purification and refolding of recombinant *Ac*CP3 protein

The correct plasmids containing *Ac*CP3 were amplified with primers containing *Hind*III and *BamH*I restriction sites, and the PCR products were ligated into the pQE-30 expression vector (Qiagen, Germany). The sequences of all constructs were confirmed on both strands and analysed with Vector NTI software (Invitrogen, Waltham, USA). Plasmids were transformed into M15 (pREP4) cells (Qiagen, Germany) for protein expression. The selected clones were cultured in Luria-Bertani broth containing 100 μg/mL ampicillin and recombinant *Ac*CP3 (rAcCP3) expression was induced using 1 mM isopropyl-β-d-thiogalactoside for 3 h at 37 °C. The recombinant r*Ac*CP3 protein was purified using a QIA Express kit in accordance with the manufacturer’s instructions. The purity and mass of protein were determined by sodium dodecyl sulphate polyacrylamide gel electrophoresis (SDS-PAGE). Since the N-terminal peptide sequence in recombinant CPs can inhibit the hydrolytic activity of the protease, it was necessary for the recombinant *Ac*CP3 to be refolded to obtain the mature peptide with hydrolytic activity [21]. Refolding of the purified recombinant protein was performed as described previously [11, 21]. In brief, purified r*Ac*CP3 (2 mg) was slowly added to 100 mL refolding buffer, containing 100 mM Tris-HCl (pH 8.0), 1 mM ethylenediaminetetracetic acid (EDTA), 250 mM L-arginine, 5 mM reduced glutathione (GST) and 1 mM oxidised glutathione. The protein was gently stirred at 4°C overnight and then dialysed against 10 mM Tris-HCl (pH 7.5). The obtained r*Ac*CP3 was further processed as described previously [22]. In brief, sodium acetate buffers with pH 4.0 to 7.0 were used to analyse the optimal pH condition required for obtaining the fully matured r*Ac*CP3 enzyme. The concentration of matured r*Ac*CP3 enzyme was measured using a protein assay (Bio-Rad, USA).

### Analysis of biological characteristics of *Ac*CP3

To assess the role of *Ac*CP3 in *Acanthamoeba* pathogenesis, the reactivation of the physiological properties of trophozoites was performed using HEp-2 cell monolayers as described previously [23, 24]. In brief, HEp-2 cells were cultured in 75 cm^2^ tissue culture flasks (Corning, USA) at 37 °C under sterile conditions until the monolayer covered the bottom of the flask completely, at which point the supernatant was removed. Trophozoites (10^6^) suspended in 25 mL physiological 0.9% NaCl were inoculated onto the monolayer three times consecutively. Cocultures of amoebae and HEp-2 cells were incubated at 26 °C until the monolayer was completely lysed. Reactivated trophozoites were collected and total mRNA was extracted. qRT-PCR was performed to analyse the expression level of *Ac*CP3. In addition, MBP was used as a virulence protein in *Acanthamoeba* [25]. 18S rDNA was used as internal reference [26]. The primers for *Ac*CP3, MBP and 18S rDNA are listed in Additional file 1: Table S2.

To determine the effect of AcCP3 on host proteins, haemoglobin (from human blood), collagen (from human placenta) and albumin (from bovine serum) were purchased from Sigma-Aldrich (USA). Each protein (2 mg/mL) was incubated with matured r*Ac*CP3 (100 nM) in 50 mM sodium acetate (pH 4.0 or pH 7.0) with 1 mM GST for 3 h at 37 °C. The reactions were terminated by adding reducing sample buffer and the degradation activity of r*Ac*CP3 was analysed by SDS-PAGE.

The protease activities of trophozoite crude proteins were analysed using Novex™ 10% Zymogram Plus (Gelatin, China) Protein Gels (Thermo Fisher Scientific, USA) in accordance with the manufacturer’s instructions, and 1.5 μg crude protein extract was added to each lane. Various inhibitors for different proteases were used. Crude protein extracts treated and untreated with inhibitors (1 h before electrophoresis) were analysed with zymography. The final concentrations of inhibitors were as follows [27]: for serine proteases, 5 mmol/L phenylmethylsulphonyl fluoride (PMSF); for metalloproteases, 2 mmol/L EDTA; for CPs, 40, 60, 80 and 100 mmol/L N-ethylmaleimide (NEM).

### AcCP3 gene silencing

siRNA targeting the catalytic domain of *Ac*CP3 was synthesised by RiboBio Co. (China) and based on the cDNA sequence. The sequence of the forward strand was 5'-AGUACAUCAUCAACAACAA-3'. Trophozoites were plated at a density of 5 × 10^4^ cells in 48-well plates, cultured overnight and then transfected with siRNA (15 μg/mL) for 12 h using SuperFectin™ In Vitro siRNA Transfection Reagent (Pufei, China). As a control, a negative siRNA provided by RiboBio Co. (China) was also applied to cultured trophozoites. Untreated trophozoites and transfection reagent-treated trophozoites were also processed. After the transfection, the differentially treated trophozoites were harvested to determine the efficacy of the knockdown by examining the expression level of *Ac*CP3 with qRT-PCR and then for the cytopathic tests.

### Effect of *Ac*CP3 knockdown in trophozoites on *Acanthamoeba*-mediated cytotoxicity

To determine the effects of reduced *Ac*CP3 expression on *Acanthamoeba*-mediated HCECs death, cytotoxicity assays were performed as previously described [28]. Confluent HCEC monolayers in 12-well culture plates (Corning, USA) were incubated with differentially treated trophozoites (ratio 1:2) at 37 °C in a 5% CO_2_ atmosphere for 24 h. Four different experimental groups were included: group 1, normal cultured confluent HCECs; group 2, HCECs co-cultured with normal trophozoites; group 3, HCECs co-cultured with negative control siRNA transfected trophozoites; group 4, HCECs co-cultured with *Ac*CP3-knockdown trophozoites. The cytopathic effects for the different groups of trophozoites were observed using light microscopy (Olympus, Japan). The trophozoites were detached with cold 2 mM EDTA-PBS buffer, chilled on ice for 20 min and then harvested. In addition, HCECs were cultured in fresh DMEM medium with CCK-8 reagent for 2 h, and the cytotoxicity was determined by measuring dehydrogenase release (Cell Counting Kit-8, DOJINDO, Japan). The absorbance of each well was measured at 450 nm using a Model 680 Microplate Reader (Bio-Rad, USA). To investigate the signalling pathways in HCECs activated by *Acanthamoeba*, four groups of differentially treated HCECs were harvested after co-culturing with amoeba for 24 h. qRT-PCR was performed to analyse the expression level of the *Ras* gene (NM_004985.4). The forward strand was 5'-AGGAAGCAAGTAGTAATTGATGGA-3'; the reverse strand was 5'-GCCTGTTTTGTGTCTACTGTTCT-3'. Human GAPDH used as an internal reference, with forward strand 5'-TCACCACCATGGAGAAGGC-3' and reverse strand 5'-GCTAAGCAGTTGGTGGTGCA-3'.

### Western blotting assays

The activation states of Raf, Erk and p53 in differentially treated HCECs were determined using Western blotting assays as previously described [29]. Briefly, four groups of differentially treated HCECs were harvested for Western blotting after coculturing with amoeba for 24 h. The cells were lysed in 100 μL lysis buffer (1 mL lysis buffer containing 20 μL phosphatase inhibitors, 20 μL protease inhibitor cocktail, 100 μL PBS, 5 μL NP-40 and 855 μL ddH_2_O). The cell lysate (20 μg) was resolved by SDS-PAGE and transferred to PVDF membranes (Roche, Switzerland). We used the following primary antibodies purchased from Cell Signaling Technology (USA): rabbit anti-p-Raf (Ser338), rabbit anti-Raf, rabbit anti-p-Erk (Thr202/Tyr204), rabbit anti-Erk, mouse anti-p-p53 (Ser15) and mouse anti-p53. HRP-labelled goat anti-mouse and goat anti-rabbit secondary antibodies (Abcam, UK) and rabbit polyclonal to β-actin (Abcam, UK) were detected with Tanon™ High-sig ECL Western Blotting Substrate (Tanon, China), observed with an ECL detection system (Tanon, China), and the scanned images were quantified using Image-Pro Plus 4.5.1 software (Media Cybernetics, USA).

### Statistical analysis

The results of qRT-PCR were calculated using the 2^−△△Ct^ method. Statistical analyses were performed using GraphPad Prism software (San Diego, CA, USA). Significance was calculated by one-way analysis of variance followed by a Tukey test or Student’s *t*-test. Data were expressed as mean ± SD and at least three independent experiments were performed for each experiment. A *P* value < 0.05 was considered significant in all analyses.

## Results

### Identification of pathogenic *Ac*CPs

The sequences encoding full-length CP genes were amplified using PCR procedures. Four new open reading frames (ORF) corresponding to CP genes were identified in *A. castellanii*. The sequences of *Ac*CP3, *Ac*CP8, *Ac*CP9 and *Ac*CP10 with the accession numbers LC472809, LC472810, LC472812 and LC472813, respectively (Additional file 1: Table S3), have been submitted to the GenBank database. *Ac*CP8 does not present the typical characteristics of CPs. The sequences of *Ac*CP6 (XM004341651) and *Ac*CP7 (XM004358251) from ATCC30011 have also been confirmed in the Neff strain.

The amino acid residues* Q, C, H* and* N* that act as the catalytic site of the cathepsin L-family CPs exist in *Ac*CPs. The modified ERFNIN and GNFD motifs that are conserved in cathepsin L-like CP family members have also been observed in *Ac*CPs [30]. We found that while *Ac*CP8 had no C active site, the amino acid sequences of the other CPs contained the conserved C site, which is mainly involved in the refolding of the protease (Additional file 1: Fig. S1). The nucleotide sequence homology of CPs between ATCC30011 and Neff 7 CPs was as high as 99%.

### Expression pattern of *Ac*CPs during encystation

**Fig. 1:**
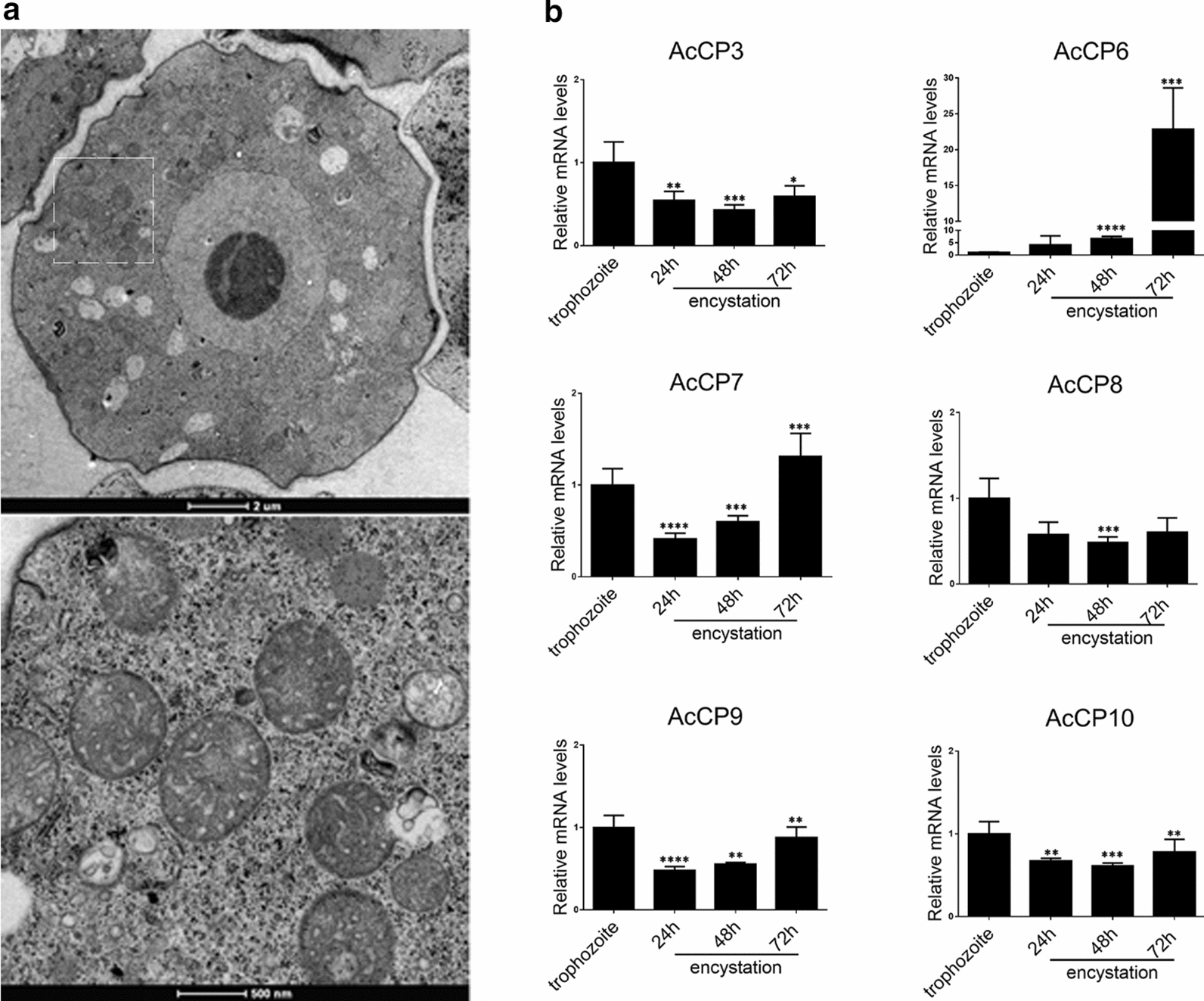
*Ac*CPs expression levels during encystation using qRT-PCR analysis.** a** Transmission electron microscopy of cysts. **b** The experiments were repeated three times, and the average values were presented with vertical bars representing standard deviations. *Means significantly different (*P* < 0.05) by Student’s* t*-test, **P < 0.01, ***P < 0.001, ****P < 0.0001

The typical structure of cysts was observed by electron microscopy after 72 h of encystment (Fig. 1a). qRT-PCR was used to analyse the expression levels of the *Ac*CPs during the encystation process. As shown in Fig. 1b, the mRNA levels of *Ac*CP3, *Ac*CP7, *Ac*CP8, *Ac*CP9 and *Ac*CP10 were decreased at 24 h, 48 h and 72 h (with the exception of *Ac*CP7 at 72 h), indicating that these *Ac*CPs may not be involved in encystation. In contrast, *Ac*CP6 was gradually but substantially up-regulated during encystation, suggesting that *Ac*CP6 may play a uniquely important role in the formation of cysts although most cathepsin L-family CPs were not involved in encystation.

### Relationship between *Ac*CPs and protozoan virulence

**Fig. 2:**
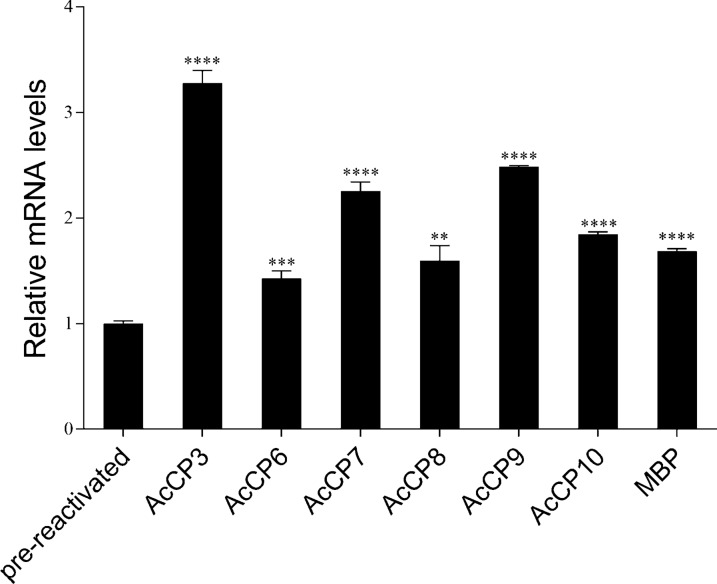
Expression levels of *Ac*CPs and MBP between pre- and post-reactivated trophozoites. *Ac*CPs include *Ac*CP3, *Ac*CP6, *Ac*CP7 *Ac*CP8, *Ac*CP9 and *Ac*CP10. Values indicate the mean (± SD) of three experiments. Student’s *t*-test was used to analyze the data. *P < 0.05, **P < 0.01, ***P < 0.001, ****P < 0.0001

To investigate the role of *Ac*CPs in *Acanthamoeba* virulence, trophozoites that were axenically grown *in vitro* for several years were reactivated with HEp-2 cells. The qRT-PCR analysis of *Ac*CP and MBP mRNA expression in pre- and post-reactivated trophozoites is shown in Fig. 2. Compared with pre-reactivated trophozoites, the MBP expression level as well as the expression levels of six of the CPs were up-regulated following trophozoite reactivation, and the expression level of *Ac*CP3 was significantly increased in trophozoites invading HEp2 cells (*P* < 0.05; Fig. 2). These data indicate that *Ac*CPs may play an important role in *Acanthamoeba* pathogenesis by mediating host cell damage during infection. Furthermore, *Ac*CP3 in particular may act as a potential pathogenic factor during *Acanthamoeba* invasion.

### Refolding of r*Ac*CP3 and degradation of host proteins

**Fig. 3:**
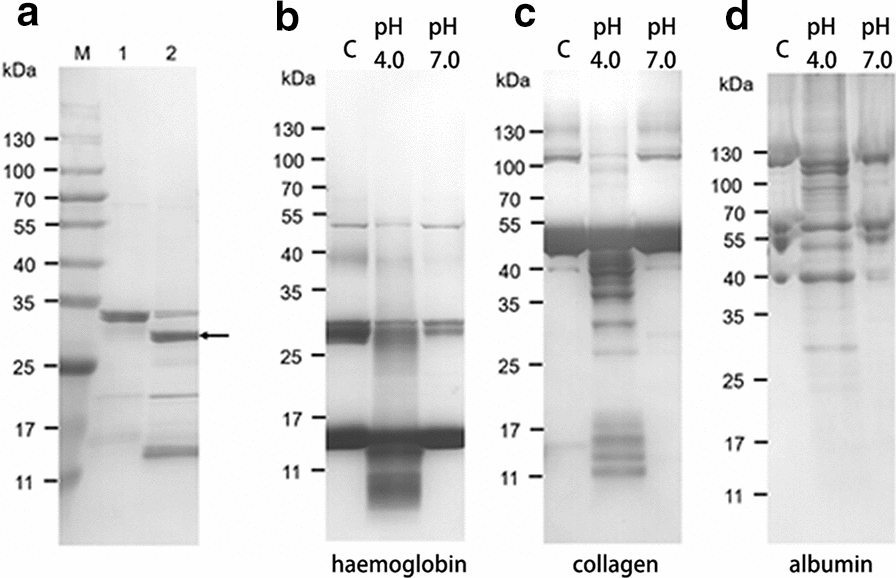
Expression, purification and refolding of r*Ac*CP3 and degradation of host proteins.** a** r*Ac*CP3 was analyzed by SDS-PAGE. Lane 1, purified r*Ac*CP3. Lane 2, refolded r*Ac*CP3. **b–d** Degradation of haemoglobin, collagen and albumin by r*Ac*CP3 in a reaction system of pH 4.0 and pH 7.0, respectively. Lane “C” mean control (without recombinant protein)

The *Ac*CP3 gene contains a 993-bp ORF encoding a 329-amino acid protein. A portion of the prodomain and the entire mature domain of *Ac*CP3 was amplified and expressed using a pQE-30 expression vector. *Ac*CP3 was expressed in M15 competent cells as an inclusion body protein with an apparent molecular mass of 34 kDa (Fig. 3a, lane 1). The recombinant protein was purified by Ni-NTA affinity chromatography and then refolded. The refolded sample was further processed under acid-reduction conditions to be processed into a fully active enzyme. The size of the fully activated mature r*Ac*CP3 was consistent with the predicted size of the mature protease (27 kDa) (Fig. 3a, lane 2). Then, the fully activated r*Ac*CP3 was used for an assay of host proteins degradation. SDS-PAGE showed that r*Ac*CP3 possesses proteolytic activity against human haemoglobin, collagen and bovine serum albumin. In addition, all these protein substrates were hydrolysed at acidic pH, but not at neutral pH (Fig. 3b–d).

### Low proteolytic activity of *Ac*CP3 knockdown trophozoites

**Fig. 4:**
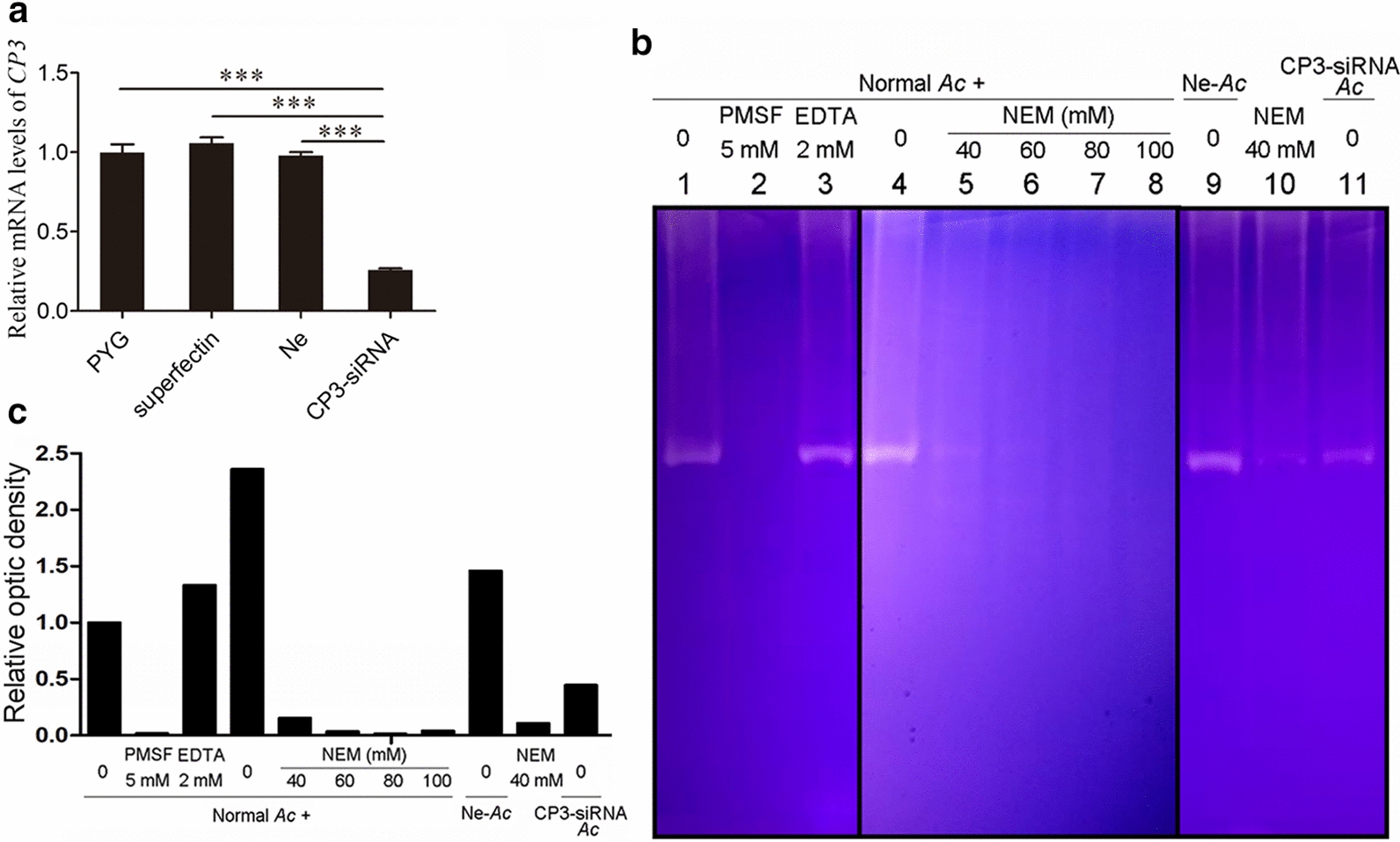
Zymography showed low hydrolytic activity of *Ac*CP3 gene knockdown trophozoites.** a** *Ac*CP3 mRNA expression level of wild-type trophozoites (PYG); only superfect-treated trophozoites (superfectin); negative siRNA transfected trophozoites; *Ac*CP3-siRNA transfected trophozoites. **b** Zymography test of different trophozoite crude extract proteins. Lane 1 and lane 4 showed normal trophozoite crudes proteins with no treatment. Lane 5 and lane 10 showed normal trophozoite crude proteins pretreated with 40 mM NEM. Lane 9 added negative siRNA transfected trophozoite crude proteins. Lane 11 added *Ac*CP3 gene knockdown trophozoite crude proteins. Independent experiments were repeated three times. **c** The optic densities of the zymography test. Significance was calculated by one-way analysis of variance (ANOVA) followed by a Tukey test. Vertical bars indicate SD. ***P < 0.001

To investigate the biological role of *Ac*CP3 in *Acanthamoeba* trophozoites, trophozoites were transfected with *Ac*CP3-siRNA. qRT-PCR revealed that the mRNA level was significantly reduced in *Ac*CP3 knockdown trophozoites (Fig. 4a). Besides, the expression of other CPs genes was not affected by *Ac*CP3-siRNA (Additional file 1: Fig. S2). There was no significant difference in the growth and proliferation rate of trophozoites between the knockdown and wild-type strains (data not shown). Furthermore, no differences observed in the growth and proliferation rate between superfectin-treated trophozoites (superfectin) and negative control siRNA transfected trophozoites (Ne) compared to untreated trophozoites cultured with PYG. Subsequently, the hydrolytic activity of *Ac*CP3 knockdown trophozoites was compared to that of wild-type trophozoites and protease inhibitor-treated trophozoites (Fig. 4b, c); 1.5 μg of differently treated crude protein extracts was added into lane 1 to lane 8 and lane 10. The crude protein extracts in lane 1 and lane 4, corresponding to wild-type trophozoites, showed high hydrolytic activity. Lane 2 and lane 3 showed that it was not the metalloprotease inhibitor EDTA but the serine proteinase inhibitor PMSF could inhibit this hydrolytic activity. Lanes 5 to 8 showed that NEM could inhibit this hydrolytic activity in a concentration-dependent manner. The ability of PMSF and NEM to inhibit the hydrolytic activity of crude protein extracts from trophozoites confirmed previous results [27]. In lane 9, 1.5 μg crude protein extract from trophozoites transfected with negative control siRNA showed comparable hydrolytic activity to that from wild-type trophozoites (lane 1 and lane 4), suggesting that the negative control siRNA has little effect on the hydrolytic activity. Lane 10 is equally treated as lane 5. Results in lane 11 showed attenuated hydrolytic activity of trophozoites transfected with *Ac*CP3 siRNA. The zymography above revealed that *Ac*CP3 was crucial for the proteolytic activity in trophozoites.

### Effect of *Ac*CP3 knockdown on protozoa-mediated cytotoxicity

**Fig. 5:**
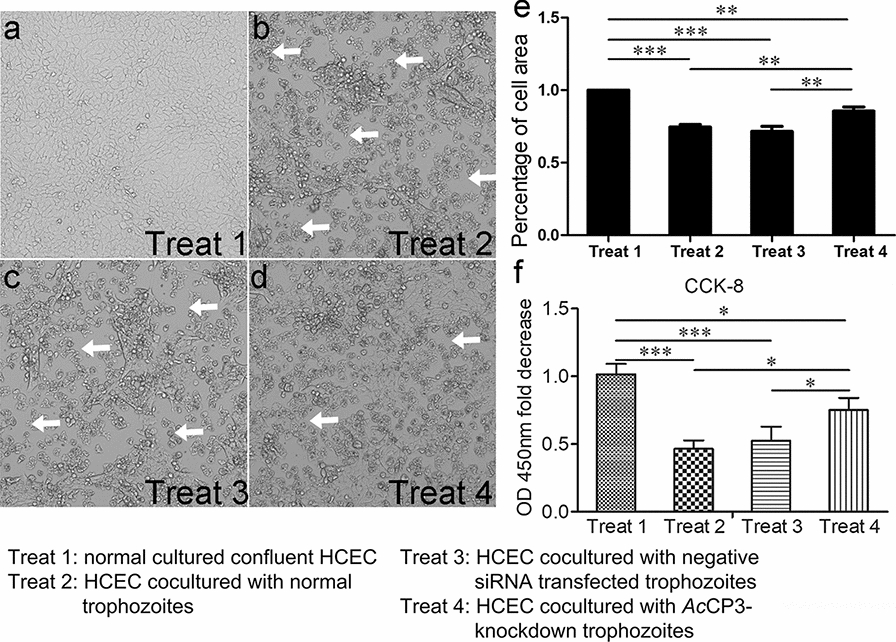
Microscopy and CCK-8 demonstrating that *Ac*CP3 gene knockdown trophozoites exhibited a decrease in *Acanthamoeba*-mediated HCEC cytotoxicity. **a–d** Pictures taken under light microscopy. **a** Normal cultured confluent HCEC (treat 1). **b** HCEC cocultured with normal trophozoites (treat 2). **c** HCEC cocultured with negative siRNA transfected trophozoites (treat 3). **d** HCEC cocultured with *Ac*CP3-knockdown trophozoites (treat 4). **e** Percentage of cell coverage to the basal area. **f** OD 450-nm-fold decrease compared to treat 1 HCEC cell. Independent experiments were repeated three times. Vertical bars indicate SD. *P < 0.05; **P < 0.01; ***P < 0.001

Cytotoxicity assays were performed with four differently treated HCECs as described in the Methods section. When HCECs were cultured alone, the cells grew into a confluent monolayer and were tightly connected to one another, with a small number of cells lifted off from the dish because of overgrowth (shown as Fig. 5a). The white arrows in Fig. 5b–d indicate the cellular voids formed when HCEC monolayer cells were destroyed by trophozoite penetration. After co-culture of confluent HCECs and wild-type trophozoites, most adherent HCECs became suspended, and the tight connections between the cells were disrupted, resulting in the formation of cellular voids (Fig. 5b), while there were no differences in the formation of cellular voids between negative control siRNA transfected trophozoites and untreated trophozoites (Fig. 5c). In addition, compared with control siRNA transfected trophozoites, trophozoites transfected with *Ac*CP3 siRNA exhibited a decrease in *Acanthamoeba*-mediated HCEC cytotoxicity, showing lesser cellular voids and more attached cells (Fig. 5c, d, e). To quantify the cytopathic activity, the dehydrogenase released from cells was detected by CCK-8 kit after co-culture of HCECs and differently treated trophozoites. After 24 h treatment, HCECs treated with *Ac*CP3 knockdown trophozoites exhibited the highest dehydrogenase release compared to HCECs treated with normal trophozoites or negative siRNA transfected trophozoites (Fig. 5f). These results indicated that *Ac*CP3 was an important pathogenic factor for *Acanthamoeba* to induce cytotoxicity of host cells.

### Inhibition of *Ac*CP3 expression antagonized Ras/Raf/Erk signalling in target cells

**Fig. 6:**
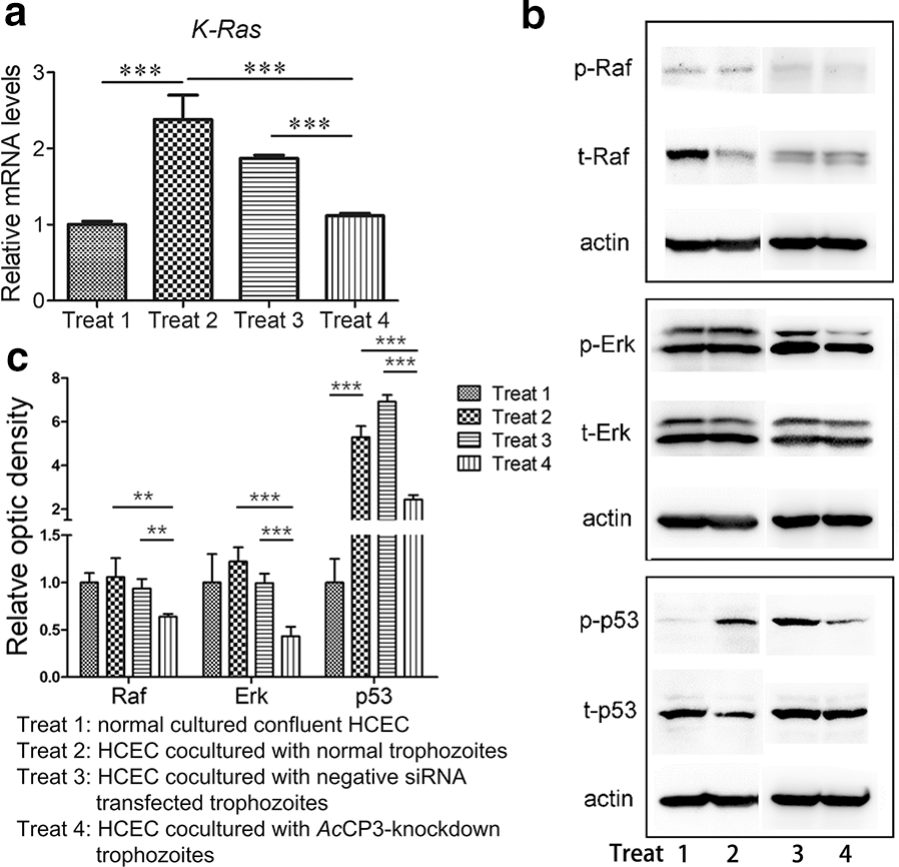
*Ac*CP3-siRNA significantly decreased the phosphorylation levels of Raf, Erk and p53 in *Acanthamoeba*-mediated HCEC cells. Four groups of HCEC were treated the same as above. **a** qRT-PCR analyzed the mRNA expression level of *K-Ras* gene. **b** Western blot analysis of the phosphorylation level of Raf, Erk and p53 proteins in HCEC cells. **c** The optic densities of phosphorylation levels of Raf, Erk and p53 proteins in HCEC cells. Normal cultured confluent HCEC (treat 1); HCEC cocultured with normal trophozoites (treat 2); HCEC cocultured with negative siRNA transfected trophozoites (treat 3); HCEC cocultured with *Ac*CP3-knockdown trophozoites (treat 4). Independent experiments were repeated three times. Vertical bars indicate SD. *P < 0.05; **P < 0.01; ***P < 0.001

Four groups of HCECs treated as described in the Methods section were harvested after co-culturing with trophozoites; qRT-PCR was used to analyse the mRNA expression levels of Ras genes. The data showed (Fig. 6a) that the expression level of K-Ras was significantly increased in HCECs co-cultured with wide-type trophozoites (treat 2). On the contrary, the expression level of K-Ras was significantly decreased in HCECs cocultured with *Ac*CP3 knockdown trophozoites (treat 4) compared with the treat 2 or treat 3 group. This indicated that *Ac*CP3 siRNA could significantly decrease the mRNA expression level of *K*-Ras in *Acanthamoeba*-treated HCECs. In contrast, the mRNA expression levels of *H*-Ras and *N*-Ras showed no differences between the treat 2 and 4 groups (data not shown). Previous studies have demonstrated that Ras recruits Raf kinase into a complex, which mediates Raf phosphorylation. Raf then phosphorylates MEK1 and MEK2, which in turn activate Erk1/2 by the tandem phosphorylation of threonine and tyrosine residues [31]. Thus, we further examined the levels of p-Raf and p-Erk in HCECs exposed to *Ac*CP3 gene knockdown trophozoites by Western blotting. These data show that untreated trophozoites could significantly increase the phosphorylation levels of Raf, Erk, and p53 proteins in HCECs (Fig. 6, treat 2). Compared to untreated cultured confluent HCECs (treat 1), HCECs co-cultured with untreated trophozoites (treat 2) or HCECs co-cultured with negative control siRNA transfected trophozoites (treat 3), the levels of p-Raf and p-Erk in HCECs co-cultured with *Ac*CP3 siRNA trophozoites (treat 4) were markedly decreased after 24 h treatment (*P* < 0.01; Fig. 6b, c). Previous studies have suggested that phosphorylation of p53 might be an important mechanism in Ras/Raf/Erk-induced apoptosis. Thus, we also examined the levels of p-p53 in HCECs cocultured with *Ac*CP3 knockdown trophozoites. The data showed that the p-p53 was also markedly down-regulated after 24 h treatment (*P* < 0.01). Results above demonstrated that inhibiting the expression of *Ac*CP3 protein could reduce the *Acanthamoeba*-mediated phosphorylation levels of Raf, Erk and p53 proteins in HCECs, suggesting that *Ac*CP3 might participate in the activation of Raf, Erk and p53 proteins in HCECs during *Acanthamoeba* invasion.

## Discussion

CP family proteins are essential to the life cycle of several protozoa by influencing several diverse processes, such as nutrient intake, protein degradation of parasites, immunomodulators, host cell invasion and encystment/excystment in addition to well-established roles in protein processing and catabolism. They are also an important virulence factor for parasites [32]. It has recently been reported that *A. castellanii* expresses CPs with proteolytic effects [11]. However, little research has been done on the pathogenic and encystment mechanisms of *Acanthamoeba* CPs. Long-term sterile passage culture of *Acanthamoeba* trophozoites *in vitro* may reduce the toxicity of the trophozoites [33]. However, a potential method to reactivate attenuated trophozoite virulence was identified by growing the trophozoites on HEp-2 cell monolayers three times using the *Acanthamoeba* 1BU strain (ATCC no. Pra-105) [23, 24]. Using this method, we found that the expression levels of six *Ac*CPs were higher following reactivation, especially *Ac*CP3, suggesting that *Ac*CPs may play a pathogenic role in *Acanthamoeba*. *In vitro*, *Ac*CP3 could effectively hydrolyse host haemoglobin, collagen and albumin after refolding, and it had strong hydrolytic activity in an acidic environment but almost no hydrolytic activity in a neutral environment. This characteristic is similar to that of vivapain from *Plasmodium vivax* [34] and CsCF-6 from *Clonorchis sinensis* [21].

The pathogenic process of *Acanthamoeba* in the host cell is regulated by various signalling molecules. G protein-coupled receptor and *β* adrenalin receptor inhibitors block the activity of trophozoite proteases, thereby affecting the growth, encystation, vitality and pathogenicity of the parasite [15]. The HCECs used in our experiments primarily express the epithelial markers E-cadherin, ZO-1 and *β*-catenin. It has been reported that Toll-like receptor 4 (TLR4) is an important pathogenic target of *Acanthamoeba* in human corneal epithelial cells (HCECs). After attaching to the surface of host cells, *Acanthamoeba* activates TLR4, which in turn affects intracellular MyD88, NF-κB and Erk [35]. It has been found that thrombinase and trypsin can activate protease-activated receptor (including PAR1 and PAR2) in HCECs and promote the secretion of pro-inflammatory factors such as IL-6, IL-8 and TNF-α, leading to ocular inflammation [36]. Furthermore, studies have shown that *Acanthamoeba-*induced host cell death is related to PI3K signalling [17]. *Acanthamoeba*-induced host cell apoptosis is associated with mitochondrial overexpression of pro-apoptotic proteins [37]. It was found that cytosolic phospholipase A2 alpha (cPLA2-*α*) is involved in *Acanthamoeba*-induced apoptosis of HCECs [18]. To determine whether *Acanthamoeba Ac*CP3 interactes with HCECs via the TLR4 receptor, it will be necessary to determine whether CPs can bind to the TLR4 receptor on the surface of HCECs as pathogen-associated molecular patterns. In this study, zymography revealed that the proteolytic activity of trophozoites was decreased following *Ac*CP3 gene knockdown, and trophozoites after *Ac*CP3 gene silencing exhibited a decrease in *Acanthamoeba*-mediated HCEC cytotoxicity. The mechanism of *Ac*CP3 in *Acanthamoeba*-mediated HCEC cytotoxicity must be confirmed regarding its role in cell signalling pathways.

The Ras/Raf/Erk signalling pathway is the main signalling pathway regulating the occurrence and development of tumours and is important in regulating apoptosis [19]. This pathway activates endogenous apoptotic pathways, such as the release of mitochondrial cytochrome c [38], as well as the activation of caspase-9 [39], caspase-8 [40] and p53 [41]. The ability of the amoeba to activate the Ras/Raf/p53 signalling pathways of host cells during infection has not been verified. Ras signalling molecules include *H*-Ras, *K*-Ras and *N*-Ras [42]. The role of Ras in HCECs has been confirmed, and it has been reported that Ras can regulate the inflammatory response in the cornea of mice [43]. In addition, the Ras/Erk signalling pathway can be activated by hepatocyte growth factor [44] in HCECs. In this study, we found that *Acanthamoeba* infection mainly affected the expression level of the *K-Ras* gene in HCECs, and Western blot analysis showed that trophozoites could also increase the phosphorylation levels of Raf, Erk1/2 and p53 in HCECs; taken together, this indicates that *Acanthamoeba* could activate the Ras/Raf/ERK/p53 signalling pathways of host cells. Moreover, studies have found that serine and cysteine protease inhibitors can down-regulate Ras pathway-induced apoptosis. Protease inhibitors can inhibit the activation of Ras, which may be related to the involvement of proteases in tumour cell invasion and metastasis [45]. In summary, proteases are involved in the activation of Ras protein and downstream signalling pathways. In this study, the phosphorylation of Ras/Raf/ERK/p53 signalling pathway components in HCECs was decreased when the expression of *Ac*CP3 was inhibited in trophozoites. This suggests that *Ac*CP3 is involved in the activation of the Ras/Raf/ERK/p53 signalling pathway during the trophozoite pathogenic process.

It is not well understood how CP expression and function change during encystation in *Acanthamoeba*. We analysed the transcription levels of six genes and found that *Acanthamoeba* CP *Ac*CP6 was the most highly expressed CP gene during the encystation process. This indicates that while most cathepsin L-family CPs are not involved in encystation, *Ac*CP6 may participate in this process. Of these CP genes, *Ac*CP6 emerges as the most highly expressed and exhibits developmental regulation, with expression increasing dramatically during encystation and cyst stages. In general, the process of encystation involves the coordinated secretion of cyst wall materials to the periphery of a cell. It was suggested that trophozoites produce abundant cyst wall proteins, including *Ac*CP6, which are packaged into encystation-specific materials in response to environmental cues. Indeed, cysteine endopeptidases in *Giardia* were localised to the encystation-specific vesicles during encystation. When fixed cysts were subjected to fluorescence in situ hybridization with *Ac*CP3 and *Ac*CP6 DNA probes, *Ac*CP3 and *Ac*CP6 were visualized in both the submembranous cytoplasm and nucleus of cysts (Additional file 1: Fig. S3). Our data indicated that some CPs could also be regulated by acidification of encystation-specific packages; although, CP3 plays a direct role in acidic vesicles, CP6 may act through other pathways and with different mechanisms, which need to be separated from the acid conditions. Both CP3 and CP6 were translated under the membrane, but there was a specific mechanism to regulate the effect of cyst formation-related CP. It had been suggested that encystation vesicles fuse with peripheral vacuoles prior to formation of the cyst wall. The activity of encystation-specific CP (*Ac*CP6) toward protein substrates is greatly reduced in an acidic compartment.

## Conclusions

These results suggested that various *Acanthamoeba* CPs may be required during pathogenic and cyst formation processes. We propose that various CPs, especially *Ac*CP3 and *Ac*CP6, play important roles in the regulation of pathogenic and encystment processes. Further study of the pathogenic mechanisms of *Acanthamoeba* trophozoites will provide a platform for the development of new anti-*Acanthamoeba* drugs. Further work will be required to clarify the role of the other CPs in *Acanthamoeba*. The present study preliminarily confirmed that *Acanthamoeba* trophozoites activate the Ras/Raf/Erk/p53 signalling pathways of HCECs and contribute to host cell death. The phosphorylation of the Ras/Raf/Erk/p53 signalling pathway decreased after inhibiting the expression of *Ac*CP3, indicating that *Ac*CP3 may be an important pathogenic factor involved in the pathogenesis of trophozoites.

## Supplementary information

**Additional file 1:** Additional figures and tables of  identification and biochemical characterisation of Acanthamoeba castellanii cysteine proteinase 3. **Figure S1.** The catalytic site of cathepsin L-family CPs exist in AcCPs. Structural organization of six A. castellanii cysteine proteases. Numbers indicate the number of amino acid residues forming the predomains, prodomains, or catalytic domains.** Figure S2. **The AcCPs expression levels of trophozoites after AcCP3 gene silencing using qRT-PCR analysis. PYG: normal cultured trophozoites, Ne: negative siRNA transfected trophozoites, and AcCP3 silencing: AcCP3-knockdown trophozoites. The average values are presented with vertical bars representing standard deviations.** Figure S3. **Location of AcCP3 and AcCP6 nucleic acids in cyst stage of Acanthamoeba using in situ hybridization. Fixed cysts were subjected to FISH with AcCP3 and AcCP6 DNA probes. AcCP3 (green) and AcCP6 (red) were visualized in both submembranous cytoplasm and nucleus of cysts (a and b). DAPI-stained cell nuclei (blue) are shown for orientation.** Table S1. **Primers used for amplification of A.castellanii genes.** Table S2.** Primers used for qRT-PCR of A.castellanii genes.** Table S3.** The information of AcCPs.

## Data Availability

All data generated or analyzed during this study are included in the article.

## Abbreviations

*Ac*CPs: *Acanthamoeba* cysteine proteases
HCECs: Human corneal epithelial cells
CP: Cysteine proteases
TLR4: Toll-like receptor-4
MyD88: TLR4-Myeloid differentiation primary response gene
NF-κB: Nuclear factor-κB
Erk: Extracellular signal-regulated kinase
PI3K: Phosphatidylinositol-3-kinase
PBS: Phosphate-buffered saline
qRT-PCR: Quantitative real-time polymerase chain reaction
SDS-PAGE: Sodium dodecyl sulphate polyacrylamide gel electrophoresis
EDTA: Ethylenediaminetetracetic acid
GST: Glutathione
PMSF: Phenylmethylsulphonyl fluoride
ORF: Open reading frames
