# Supplementary material for: Identification and biochemical characterisation of Acanthamoeba castellanii cysteine protease 3
Source: Parasit Vectors. 2020 Nov 23;13:592. doi: 10.1186/s13071-020-04474-8 (PMC7685649; doi:10.1186/s13071-020-04474-8)
Supplement: Supplementary file 1 — Additional file 1: Additional figures and tables of identification and biochemical characterisation of Acanthamoeba castellanii cysteine proteinase 3. Figure S1. The catalytic site of cathepsin L-family CPs exist in AcCPs. Structural organization of six A. castellanii cysteine proteases. Numbers indicate the number of amino acid residues forming the predomains, prodomains, or catalytic domains. Figure S2. The AcCPs expression levels of trophozoites after AcCP3 gene silencing using qRT-PCR analysis. PYG: normal cultured trophozoites, Ne: negative siRNA transfected trophozoites, and AcCP3 silencing: AcCP3-knockdown trophozoites. The average values are presented with vertical bars representing standard deviations. Figure S3. Location of AcCP3 and AcCP6 nucleic acids in cyst stage of Acanthamoeba using in situ hybridization. Fixed cysts were subjected to FISH with AcCP3 and AcCP6 DNA probes. AcCP3 (green) and AcCP6 (red) were visualized in both submembranous cytoplasm and nucleus of cysts (a and b). DAPI-stained cell nuclei (blue) are shown for orientation. Table S1. Primers used for amplification of A.castellanii genes. Table S2. Primers used for qRT-PCR of A.castellanii genes. Table S3. The information of AcCPs. [file 13071_2020_4474_MOESM1_ESM.pdf]

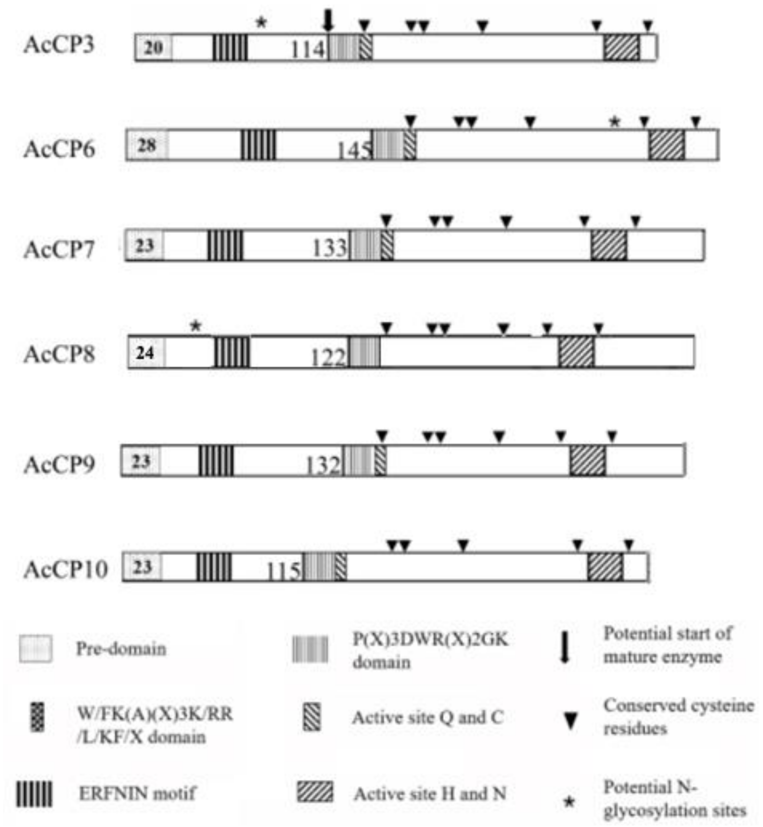

Figure S1. The catalytic site of cathepsin L-family CPs exist in *Ac*CPs. Structural organization of six *A. castellanii* cysteine proteases. Numbers indicate the number of amino acid residues forming the predomains, prodomains, or catalytic domains.

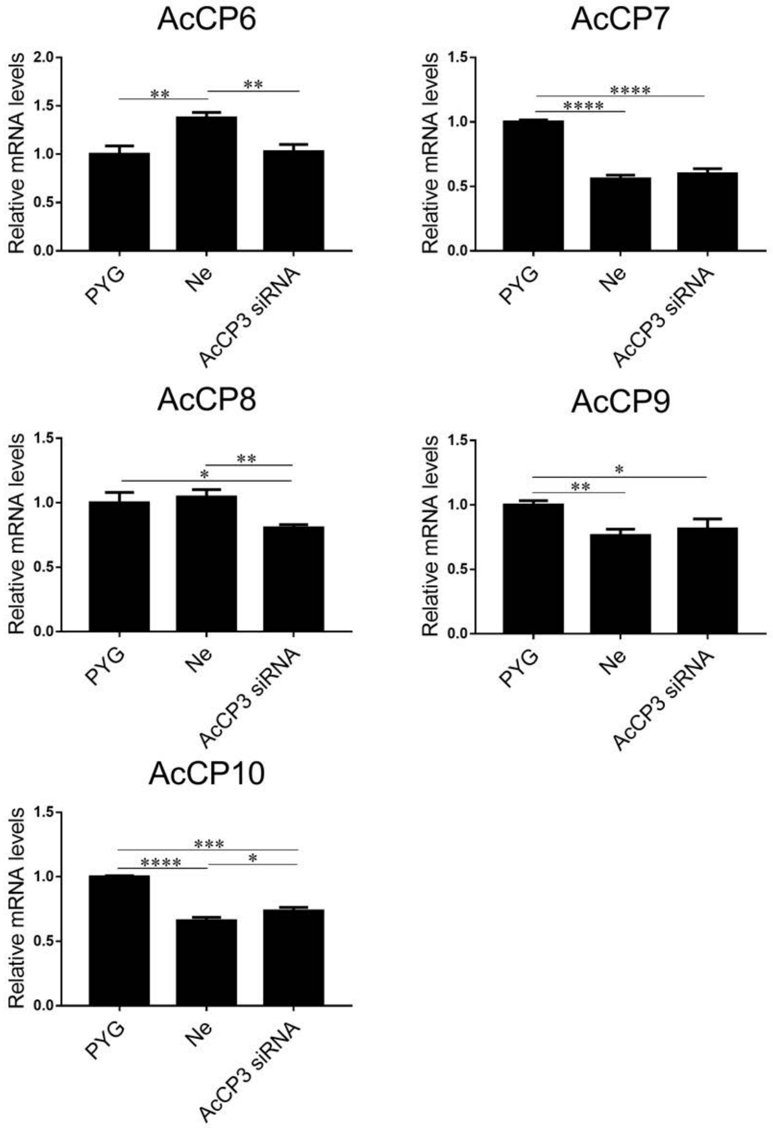

Figure S2. The *Ac*CPs expression levels of trophozoites after *Ac*CP3 gene silencing using qRT-PCR analysis. PYG: normal cultured trophozoites, Ne: negative siRNA transfected trophozoites, and *Ac*CP3 silencing: *Ac*CP3-knockdown trophozoites. The average values are presented with vertical bars representing standard deviations.

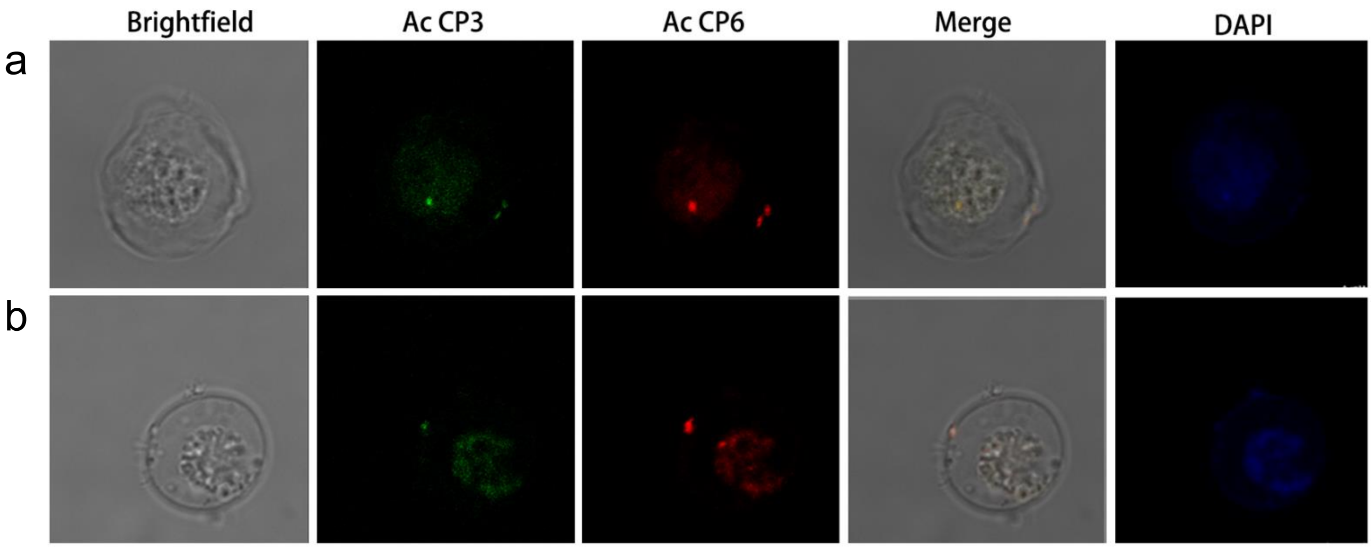

Figure S3. Location of *AcCP3* and *AcCP6* nucleic acids in cyst stage of *Acanthamoeba* using *in situ* hybridization. Fixed cysts were subjected to FISH with *Ac*CP3 and *Ac*CP6 DNA probes. *Ac*CP3 (green) and *Ac*CP6 (red) were visualized in both submembranous and nucleus of cysts (**a** and **b**). DAPI-stained cell nuclei (blue) are shown for orientation.

Methods: Fluorescence *in situ* hybridization (FISH)

Cysts of *Acanthamoeba* were fixed in 4%PFA-PBS, then treated with Proteinase K and hydrochloric acid. Cysts were washed with DEPC-PBS and incubated with fluorescence labeled *Ac*CP3 probe (FAM-5’-GGCGAGGGTAGAGGCCACGCAGAT GGCGGCAAGCAGGACGAGGATGGTGATGGCGCGCAT-3’) and *Ac*CP6 probe (TAMRA-5’-TCGTCGAAGCGCGGGCTGATGGCCGATCCCAGGGTCGCCAGG CACAGCACCACGCAGGCC-3’) at 60°C for 24 h. Cyst then were rinsed in washing buffer and stained with DAPI dye. Finally, cysts were mounted and viewed under a laser confocal microscope.

Table S1. Primers used for amplification of *A.castellanii* genes

gene	Sense primer(5’-3’)	Antisense primer (5’-3’)
CP3	ATGCGCGCTACCACCATCCTCG	TTAAGCGGTGGGGTACGAGGC
CP6	ATGAAGATGAACAAGATGACGA	TTACTGGGAGCAATCGGCCTT
CP7	ATGAAGAGCGCCACCCTTTTGT	TTAGACGATGGGGTAGGAGTTG
CP8	ATGCTGCACCCCACCACCGCTCT	TTAAACGACGACGGGATAGGAGGC
CP9	ATGAGGGTCTCTATCTTTTTGT	GACGGGGTAGGAGACATCGTAG
CP10	ATGGCGCGCGTCCACTACGTT	TTACACGAGGGGAATGGTCAC

Table S2. Primers used for qRT-PCR of *A.castellanii* genes

gene	Sense primer(5'-3')	Antisense primer (5'-3')
CP3	CGATGCCTCGCACAACTCCTTC	CCACGAGTTCTTGACGAGCCA
CP6	AGCTCACCTCCCTCTCCGAA	GATCTCCATGTTGCCTCCGT
CP7	CTTCTCCCACACCAGGACCCC	GTCCCTGCGTGATGATAGCG
CP8	CGTCCACAATGCCACCTCCTC	GAACGTAGTGCCCCCACTTCG
CP9	GTGCTCCTTCAACACCACCCG	AGCCGTCAAAGACACCAGACT
CP10	CCAGGGCAACGGCGACTACGGCT	CGGCAGAGGGCTTGAAGGCACAC
MBP	GTGAACGCGCAATATGTC	ACGGGTGAAGGTGAAGTA
GAPDH [1, 2]	AGCAGCAAACACCACTCTCACGAA	ACACCATGTAGTCCGACGCTCATG
18s rDNA	TCCAATTTTCTGCCACCGAA	ATCATTACCCTAGTCCTCGCGC

Table S3. The information of *Ac*CPs

Genbank Accesion number		Number in this article
XM004341651	Acanthamoeba castellanii str. Neff papain family cysteine protease subfamily protein (ACA1_198270) mRNA, complete cds	*Ac*CP6
XM004358251	Acanthamoeba castellanii str. Neff papain family cysteine protease subfamily protein (ACA1_018960) mRNA, complete cds	*Ac*CP7
LC472809.1	Acanthamoeba castellanii ATCC 30011 mRNA for cysteine protease 3	*Ac*CP3
LC472810	Acanthamoeba castellanii ATCC 30011 mRNA for cathepsin L 2 precursor	*Ac*CP8
LC472812.1	Acanthamoeba castellanii ATCC 30011 mRNA for papain family cysteine protease containing protein	*Ac*CP9
LC472813	Acanthamoeba castellanii ATCC 30011 mRNA for cysteine proteinase precursor	*Ac*CP10

Supplementary reference

1. Krtková J, Thomas EB, Alas GC, Schraner EM, Behjatnia HR, Hehl AB, Paredez AR. Rac regulates *Giardia lamblia* encystation by coordinating cyst wall protein trafficking and secretion. mBio. 2016; 7: e01003-16.
2. Köhsler M, Leitsch D, Müller N, Walochnik J. Validation of reference genes for the normalization of RT-qPCR gene expression in *Acanthamoeba* spp. Sci Rep. 2020; 10: 10362.
